# Retinoschisis and Norrie disease: a missing link

**DOI:** 10.1186/s13104-021-05617-5

**Published:** 2021-05-26

**Authors:** Rahini Rajendran, Dhandayuthapani Sudha, Subbulakshmi Chidambaram, Hemavathy Nagarajan, Umashankar Vetrivel, Jayamuruga Pandian Arunachalam

**Affiliations:** 1 Present Address: Central Inter-Disciplinary Research Facility (CIDRF), Sri Balaji Vidyapeeth (Deemed To Be University), Mahatma Gandhi Medical College and Research Institute Campus, Pondicherry, 607402 India; 2 SN ONGC Department of Genetics and Molecular Biology, Vision Research Foundation, Nungambakkam, Chennai, 600006 India; 3 Department of Biochemistry and Molecular Biology, Pondicherry University, Puducherry, 605014 India; 4 Kamalnayan Bajaj Institute for Research in Vision and Ophthalmology, Vision Research Foundation, Sankara Nethralaya, Chennai, 600006 India; 5 National Institute of Traditional Medicine, Indian Council of Medical Research, Belagavi, 590010 India

**Keywords:** RS1, NDP, Protein–protein interaction, MALDI-TOF mass spectrometry, Functional association

## Abstract

**Objective:**

Retinoschisis and Norrie disease are X-linked recessive retinal disorders caused by mutations in *RS1* and *NDP* genes respectively. Both are likely to be monogenic and no locus heterogeneity has been reported. However, there are reports showing overlapping features of Norrie disease and retinoschisis in a *NDP* knock-out mouse model and also the involvement of both the genes in retinoschisis patients. Yet, the exact molecular relationships between the two disorders have still not been understood. The study investigated the association between retinoschisin (RS1) and norrin (NDP) using in vitro and in silico approaches. Specific protein–protein interaction between RS1 and NDP was analyzed in human retina by co-immunoprecipitation assay and MALDI-TOF mass spectrometry. STRING database was used to explore the functional relationship.

**Result:**

Co-immunoprecipitation demonstrated lack of a direct interaction between RS1 and NDP and was further substantiated by mass spectrometry. However, STRING revealed a potential indirect functional association between the two proteins. Progressively, our analyses indicate that FZD4 protein interactome via PLIN2 as well as the MAP kinase signaling pathway to be a likely link bridging the functional relationship between retinoschisis and Norrie disease.

**Supplementary Information:**

The online version contains supplementary material available at 10.1186/s13104-021-05617-5.

## Introduction

X-linked retinoschisis (XLRS) is a retinal disorder caused by mutations in *RS1* gene (encoding retinoschisin) leading to splitting of retinal layers which impairs visual signal processing [1, 2]. Retinoschisin (24-KDa) is a cell adhesion secretory protein which helps in maintaining structural and functional integrity of retina [3, 4]. RS1 is expressed mainly in photoreceptors and bipolar cells of the retina [2, 3] and also in pinealocytes [5]. Retinoschisis is rarely known to be associated with other ophthalmic disorders like Best disease, leukocoria, neovascular glaucoma and Coats’ disease, which are different ocular entities manifesting in the same eye [6–9].

Norrie disease (ND) is an X-linked recessive disorder, characterized by ocular dysgenesis, progressive mental retardation, and deafness [10]. *NDP* (Norrie disease pseudoglioma) is the gene implicated in the disorder and it accounts for a number of variations in the affected individuals [11, 12]. *NDP* encodes a small secretory protein termed Norrin (15-KDa), with limited expression in the brain, retina and olfactory bulb and it is assumed to be involved in neurogenesis and cell–cell interaction [13, 14]. Rarely variations in the *NDP* gene are known to cause diverse forms of *NDP*-related retinopathies such as Coat’s disease, X-linked familial exudative retinopathy, retinopathy of prematurity and persistent hyperplastic primary vitreous [15–18].

Though genetic loci for retinoschisis (Xp22.13) and Norrie disease (Xp1.3) are distinct from each other, a knock-out mouse model of ND has been shown to exhibit retinoschisis-like alterations [2, 11, 19]. There is also a report on familial retinoschisis patients harboring digenic variations in *RS1* and *NDP* genes, yet, segregating only with XLRS pathology [20]. It is further intriguing to note that certain ocular features like retrolental fibrovascular membrane, retinal traction, and retinal detachment are common features of both the disorders [21]*.* These findings might be indicative of an unknown association or interaction between RS1 and NDP.

To understand the biological basis of pathogenesis and to subsequently develop methods for prevention and treatment, it is necessary to identify the molecules and the mechanisms triggering, participating, and controlling the disease process. In many disorders, protein–protein interaction (PPI) networks are being explored as there exists a complex interplay between disease genes [22, 23]. So far, only few specific RS1 binding partners have been identified such as Na^+^/K^+^ ATPase, SARM1, alphaB crystalline, beta2 laminin and L-type voltage-gated calcium channel [24–26]. Likewise, NDP has been shown to interact with leucine rich repeat containing G protein-coupled receptor 4, frizzled class receptor 4 (FZD4), LDL receptor related protein 5 and tetraspanin 12 [27–30]. There is no comprehensive study on the complete interactome of RS1 or NDP, which might provide insights into the unknown functional role of the two proteins. Therefore, we were interested to assess the physical interaction between RS1 and NDP in human retinal tissue and investigate the molecular network of RS1 and NDP to show the functional relationship.

## Main text

### Methods

Retinal tissue was isolated from human donor eyes (50 to 60 years old) with no history of ocular morbidities from CU Shah Eye bank, Sankara Nethralaya, Chennai, India. To determine physical interaction between RS1 and NDP, co-immunoprecipitation was performed in the human retinal tissue and to further investigate the complete interactome of retinoschisin and norrin, the immunoprecipitated complexes were analyzed by peptide mass fingerprinting. The resulting peptide spectra were analysed through the MASCOT search engine against Swissprot database [31]. Gene Ontology (GO) based functional annotation and enrichment analysis of data sets; FunRich (Functional Enrichment Analysis Tool) and STRING (Search Tool for the Retrieval of Interacting Genes/Proteins) were used to predict the functional association between the two target proteins along with the interactomes [32, 33].

A detailed methodology section is provided as additional file 1.

### Results

#### Protein–protein interaction between RS1 and NDP

**Fig. 1:**
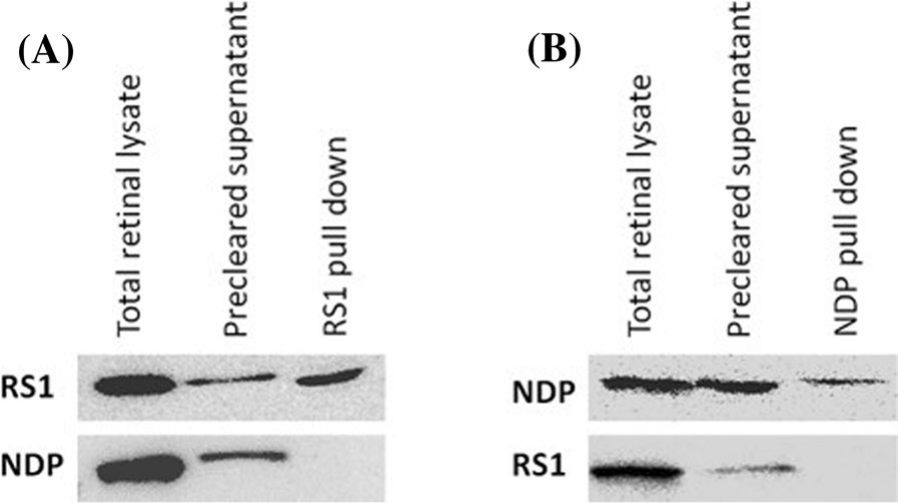
**A** Immunoblot images of RS1 co-immunoprecipitation assay showing the presence of retinoschisin, but the absence of norrin in the immunoprecipitated (IP) fraction. **B** Immunoblot images of NDP co-immunoprecipitation assay showing the presence of norrin, but the absence of retinoschisin in the immunoprecipitated (IP) fraction

The physical association between RS1 and NDP was investigated in the human retina using co-immunoprecipitation assay. On immunoblotting, RS1 was identified in the RS1 immunoprecipitated complex, while NDP was not detected (Fig. 1A). Likewise, in the NDP immunoprecipitated fraction, NDP was found, while RS1 was not detected (Fig. 1B). These in vitro experiments revealed that RS1 and NDP did not exhibit any physical interaction as evident from Fig. 1.

#### Identifying the putative binding partners of RS1 and NDP

To validate the co-immunoprecipitation results, the immunoprecipitated complex of RS1 and NDP were individually analyzed by MALDI-TOF mass spectrometry. The resultant MS spectra did not detect NDP in the RS1 immunoprecipitated complex and, RS1 was not identified in the NDP complex. Further, to understand the functional relationship, we scrutinized the MS spectra of RS1 and NDP complex for other potential binding partners. Though the MASCOT analysis identified 190 genes in RS1 sample and 159 genes in NDP sample, most of the protein hits were below the threshold protein score (p < 0.05). The complete lists of putative RS1 and NDP binding proteins are provided in the Additional file 2, 3 (Table S1 and S2). ACTB (Beta-Actin) was exclusively found to have a significant protein score of 72 and 48 in the RS1 and NDP immunoprecipitated complex respectively.

**Fig. 2:**
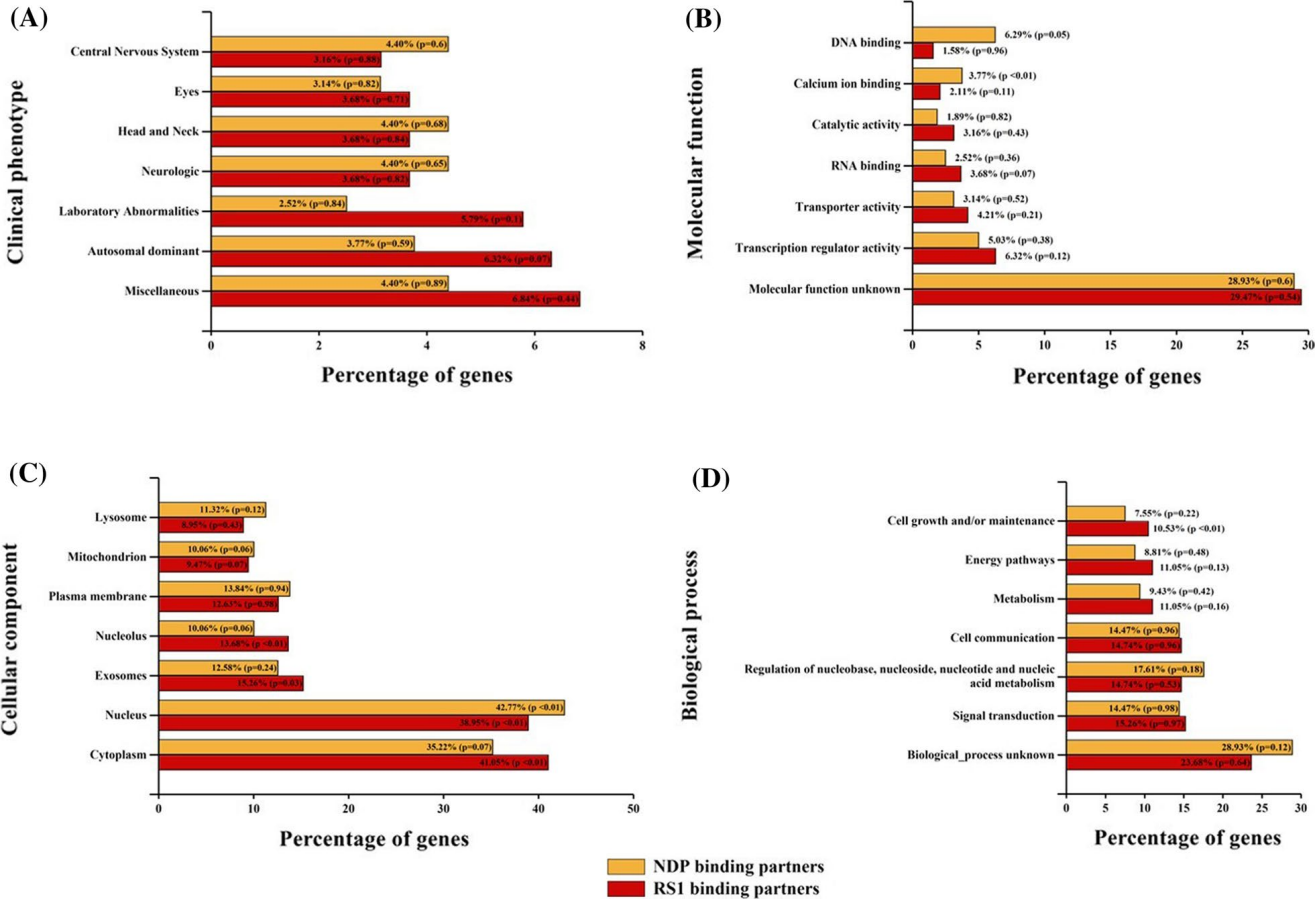
Gene ontology-based categorization of proteins identified in the immunoprecipitated complex of RS1 and NDP, analyzed by MALDI-TOF mass spectrometry. **A** Clinical phenotype. **B** Molecular function. **C** Cellular component. **D** Biological process

Gene ontology-based categorization and functional annotation using FunRich revealed that RS1 and NDP binding partners were significantly enriched in the biological process of signal transduction and cell communication (29.9% and 28.8% respectively). On the basis of cellular compartment, it was observed that a majority of the RS1 and NDP associating proteins were localized to the cytoplasm (41% and 35.2% respectively). Classification based on clinical phenotype showed that the proteins were involved in neurological, eye and central nervous system functions. The distribution of proteins under each category is represented in Fig. 2.

#### Functional protein–protein interaction network prediction

**Fig. 3:**
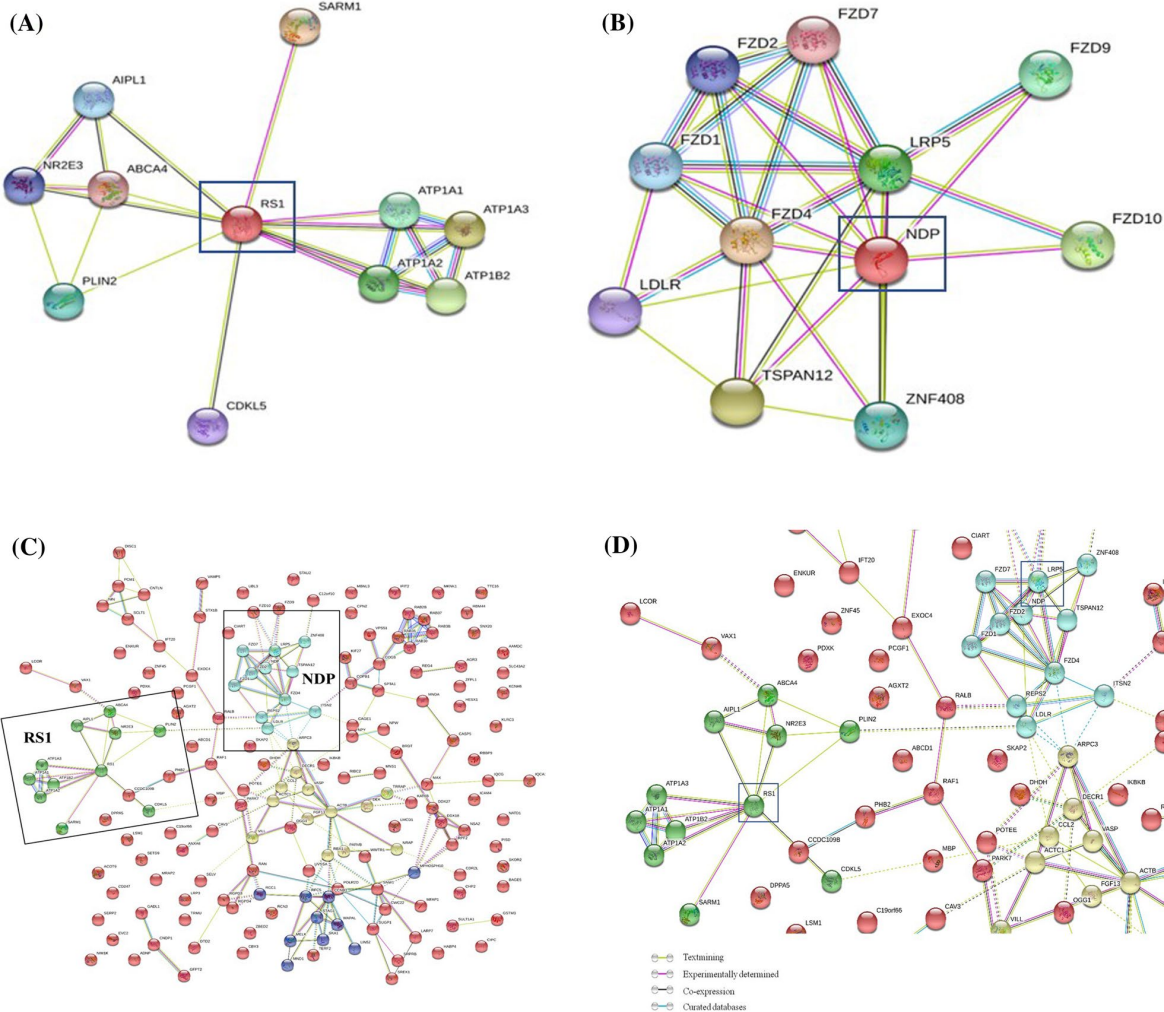
Interaction network map of RS1 and NDP generated using STRING. In the PPI network graphs, the nodes represent the proteins and the lines connecting them represent the interactions between them. **A** PPI of Retinoschisin. **B** PPI of Norrin. **C** PPI showing the association between retinoschisin and norrin. **D** The network of proteins focussed on the major interactions between RS1 and NDP, inter-cluster edges are represented by dashed-lines

With STRING database, the network map individually derived for RS1 and NDP showed proteins representing the first shell of interactors which included genes derived from text mining as well as experimental evidences and no direct protein interactions were observed between RS1 and NDP (Fig. 3A, B). As the aim of the study was to learn primarily about the functional association between RS1 and NDP, a network map was established feeding RS1and NDP as the query input genes (Fig. 3C, D).

Accordingly, 169 and 200 proteins found to be interacting with RS1 and NDP respectively, from the MALDI analysis along with the already known interactions of the respective proteins were processed further for network analysis. From this processed protein network (Fig. 3C, D), it was observed that hypothetically RS1 and NDP would crosstalk indirectly by means of Perilipin-2, or Calcium uniporter regulatory subunit MCUb (CCDC109B). In case of the interaction via Perilipin (Combine score: 0.621), it mainly contributed putatively by Low-density lipoprotein receptor that leads to FZD4 (Combine score-0.971) and NDP. On the other hand, via CCDC109B, RS1 indirectly interacts by means of Prohibitin-2—RAF (proto-oncogene serine/threonine-protein kinase) (Combined Score: 0.610)—Ras-related protein Ral-B and FZD4 (Combined Score: 0.594) leading to crosstalk with NDP (Combined Score: 0.997).

## Discussion

A study on protein interactions is fundamental to the understanding of biological systems and disease mechanism. Deciphering protein interactomes is a challenging task due to the dynamic nature of the protein–protein interactions and the fact that they are highly cell-state specific [34]. Accordingly, while literature suggest the existence of digenic involvement of *RS1* and *NDP* in the pathophysiology of retinoschisis and Norrie disease, ours is a preliminary study wherein, apart from in vitro experiments using human retinal samples, we have used in silico approaches using open access protein–protein interaction databases that provide information derived from multiple sources like experimental findings, computational predictions, mining of other databases and literature [35], to determine the association between the two proteins of interest, RS1 and NDP.

Many PPI based association between disorders have been proposed with the assumption that network neighbour gene of a target disease gene is likely to be involved in a specific biological process causing a similar disease or phenotype [36]. Based on this concept, we have correlated the MS data with the STRING predicted PPI network to establish the link between retinoschisis and Norrie disease. Of note, 23 candidate proteins were identified in the immunoprecipitated complex of both RS1 as well as NDP, though with less protein score. Representative common candidate proteins include ubiquitin thioesterase otulin, myoglobin, C-type lectin domain family 2 member B, dual specificity protein phosphatase 22, humanin-like 3, hipocalcin-like protein 4, wings apart-like protein etc. The list of common proteins and their respective biological significance is provided in Additional file 4: Table S3.

Our data show that though NDP and RS1 were not directly interacting, there might be an indirect interaction through FZD4. NDP binds with extracellular cysteine rich domain of FZD4 receptor and β-propeller of LDLR 5/6 thus activating canonical β-catenin signaling which plays a role in eye development and angiogenesis [37]. Mutation in the LDLR5 receptor and FZD4 causes Familial Exudative Vitreoretinopathy (FEVR). PLIN2 directly interacts with the lipid molecule and helps in the structure of phosphatidylcholine and sphingomyelin supporting the function of cholesterol and fatty acid. LDLR promotes the endocytosis of cholesterol-rich LDL and maintains the LDL level in plasma [38]. However, there is no experimental evidence that specified the direct interaction between PLIN2 and LDLR. But studies stated that overexpression of PLIN2 resulted in a decrease of 3-Hydroxy-3-Methylglutaryl-CoA Reductase with no decrease in LDLR [39]. Correspondingly, retinoschisin binds negatively charged membrane lipids, such as phosphatidylserine as well as phosphoinositides and might associate with Perilipin 2. This correlates with the functional role of retinoschisin in facilitating cell–cell adhesion processes in the retina, via homomeric interaction between its octamers present on the surface of two neighboring cells which is required for normal structure and function of the retina. Taken together, FZD4 via PLIN2 may act as the crucial point for the indirect interaction of NDP to RS1.

It is also noteworthy to mention that MAPK Erk1/2 pathway has been reported to be the predominant pathway implicated in the pathogenesis of retinoschisis as well as Norrie disease knock-out mouse models [40, 41]. In addition, a recent study has demonstrated the role of RS1 in regulating MAP kinase signaling and apoptosis in the retina [42]. Supporting this finding, many proteins (RAF proto-oncogene serine/threonine-protein kinase, Phosphatidylinositol 5-phosphate 4-kinase, MAP kinase-interacting serine/threonine-protein kinase, Ras-related protein Rab) involved in the MAPK signaling pathway were detected in the immunoprecipitated complex of RS1 and NDP [43–45].

Nevertheless, there may be likely incidences where open access databases might miss crucial data obtained from experimental evidence. Likewise, in vitro techniques such as MALDI-TOF mass spectrometry may fail to provide a complete set of interacting proteins due to its detection limits. Hence it is necessary to validate and characterize these proteins more extensively in order to understand the true functional relationship between the two disorders.

Despite the fact that the proteins identified by immunoprecipitation coupled with MS analyses did not exhibit a good protein score due to stringent analysis criteria, we sought to identify the probable and putative binding partners of the two target proteins.

Furthermore, it was interesting to correlate our immunoprecipitated MS data with the microarray based differential gene expression analysis of a retinoschisis and Norrie disease knock-out mouse models individually. Several proteins detected in the RS1 immunoprecipitated complex were reported to be either upregulated or downregulated in the RS1 deficient retina [40]. With reference to NDP, we found IQ domain containing protein, gamma-aminobutyric acid receptor and actin in the upregulated list of genes, while solute carrier family and zinc finger protein were among the downregulated genes in *NDP* knock-out mice [41]. This information might help in understanding the complex interaction network of RS1 and NDP.

## Conclusion

Based on our findings and analyses, we conclude that RS1 and NDP do not involve in a direct protein–protein interaction. Though our results provide evidence for the lack of a physical interaction, elaborate investigation and the possible indirect functional association needs to be carried out. PPI analysis has indicated that FZD4 protein interactome via PLIN2 as well as the MAP kinase signaling pathway to be a likely link bridging the functional relationship between retinoschisis and Norrie disease.

## Limitations

Any conclusions derived based on in vitro as well as in silico PPI methods needs to be validated since these approaches are subjected to their own limitations. The results obtained has prompted us to study the complete immunoprecipitated complex of RS1 as well as NDP using more advanced mass spectrometric platforms, which might serve as a template for future investigations on the interaction network of RS1 or NDP.

## Supplementary Information

**Additional file 1:** Detailed methodology.

**Additional file 2:** **Table** **S1.** Complete list of proteins identified in the RS1 immunoprecipitated complex analyzed by MALDI-TOF mass spectrometry.

**Additional file 3:** **Table** **S2.** Complete list of proteins identified in the NDP immunoprecipitated complex analyzed by MALDI-TOF mass spectrometry.

**Additional file 4:** **Table** **S3.** List of common proteins identified in both RS1 and NDP MALDI-TOF mass spectrometry data.

## Data Availability

The datasets on RS1 and NDP binding proteins as well as the list of common proteins along with their respective biological significance are presented as supplementary spreadsheets.

## Abbreviations

RS1: Retinoschisin
NDP: Norrie disease pseudoglioma
XLRS: X-linked retinoschisis
ND: Norrie disease
PPI: Protein–protein interaction
MS: Mass spectrometry
GO: Gene ontology
FunRich: Functional enrichment analysis tool
STRING: Search tool for the retrieval of interacting genes/proteins
ACTB: Beta actin
FZD4: Frizzled class receptor 4
PLIN2: Perilipin 2
MAP kinase: Mitogen-activated protein kinase
LDLR 5/6: Low-density lipoprotein receptor related protein 5/6
FEVR: Familial exudative vitreoretinopathy
