# Supplementary material for: Retinoschisis and Norrie disease: a missing link
Source: BMC Res Notes. 2021 May 26;14:204. doi: 10.1186/s13104-021-05617-5 (PMC8157631; doi:10.1186/s13104-021-05617-5)
Supplement: Supplementary file 1 — Additional file 1: Detailed methodology. [file 13104_2021_5617_MOESM1_ESM.pdf]

Detailed Methodology

Immunoprecipitation assay and immunoblotting

Retinal tissue was isolated from human donor eyes (50 to 60 years old) with no history of ocular morbidities from CU Shah Eye bank, Sankara Nethralaya, Chennai, India. The tissue was washed with phosphate buffered saline (PBS) twice and homogenized using lysis buffer (25mM Tris Cl (pH 7.6), 150mM sodium chloride, 1% Triton X-100, 0.1% Sodium dodecyl sulphate and 0.5% Sodium glyoxycholate). The extract was centrifuged for 10 min at 3000g to remove cell debris. The supernatant was precleared with protein A/G-agarose beads (Santa Cruz Biotechnology, Dallas, Texas, USA) by rocking it for 1 hour at 4°C to avoid nonspecific binding. The beads were spun down and the supernatant was incubated with 15µl of polyclonal anti-retinoschisin antibody (Santa Cruz Biotechnology, Dallas, Texas, USA) for overnight at 4°C in agitation on rotating rocker. Following antibody incubation, 30µl of protein A/G-agarose beads were added and incubated for another 4 hours at 4°C which was then centrifuged to separate the beads from the supernatant. The beads were washed twice with lysis buffer and thrice with PBS. Then the protein complex was eluted by allowing it to boil for 10 min in 4X Laemmli buffer. The RS1 immunoprecipitated complex was assessed for NDP protein by immunoblotting using polyclonal anti-norrin antibody (Santa Cruz Biotechnology, Dallas, Texas, USA). The same protocol was applied to pull down NDP protein immune complex using anti- norrin antibody and anti-retinoschisin antibody was used in the western blot analysis to probe RS1 and substantiate the protein-protein interaction.

Peptide mass fingerprinting and protein identification

The eluted immunoprecipitated complex of retinoschisin was separated on a 12% SDS-PAGE gel, which was then stained using Coomassie brilliant blue. Prominent bands were excised from the gel and the proteins in the gel pieces were reduced by adding 10mM dithiothreitol and incubating for 45 min at 56°C. Followed by the alkylation of the protein using 55mM iodoacetamide for 30 min at room temperature in the dark, they were then trypsin digested for 45 min at 4°C. The gel pieces were then immersed in ammonium bicarbonate and incubated overnight at 37°C. Afterwards, the peptides from each gel piece were extracted using 80% acetonitrile and 0.5% formic acid. The extracted peptides were further concentrated and desalted using Zip Tips (Millipore Corporation, Bedford, USA). The sample was applied on the target plate and mixed with the matrix solution (α-Cyano-4-hydroxycinnamic acid in 50% acetonitrile and 0.1% trifluoroacetic acid) at a ratio of 1:2. The mixture was allowed to air dry and then MALDI-TOF mass spectrometry (MS) analysis was performed using Bruker's autoflex speed TOF/TOF MS/MS system (Bruker, Billerica, Massachusetts, USA) at Shrimpex Biotech Services, Chennai, India. MALDI-TOF MS was operated at accelerating 20kV and the mass spectra were acquired in reflector positive ion mode with laser intensity set to 2500 according to the manufacturer's instructions.

MASCOT search engine against Swissprot database was used to analyze the peptide spectra obtained [1]. The parameters set were: carbamidomethylation of cysteine as fixed modification; protein N-terminal acetylation, deamidation of asparagine and glutamine, and oxidation of methionine were set as variable modifications; trypsin was used as protease with maximum 1 missed cleavage allowed.

Bioinformatics analysis

Gene Ontology (GO) is widely used in functional annotation and enrichment analysis of data sets. GO based categorization of the RS1 and NDP pull-down complex were performed using FunRich (Functional Enrichment Analysis Tool) [2]. An open access database, STRING (Search Tool for the Retrieval of Interacting Genes/Proteins) was used to predict the functional association between the two target proteins. These PPI predictions might help in understanding the relationship between the two disorders, based on the fact that a specific functional interaction between the two proteins likely contributes to a common biological event. STRING version 11.0 hosts a collection of known as well as predicted protein-protein interactions, gathered from experimental data, computational prediction and text mining [3]. In our study, the protein-protein interaction networks of RS1 and NDP were generated with a medium confidence score of 0.4.

References

1. Perkins D, Pappin D, Creasy D, Cottrell J. Probability-based protein identification by searching sequence databases using mass spectrometry data. Electrophoresis. 1999;20: 3551–3567. DOI: 10.1002/(SICI)1522-2683(19991201)20:18<3551::AID-ELPS3551>3.0.CO;2-2 PMID: 10612281

2. Pathan M, Keerthikumar S, Ang C, Gangoda L, Quek C, Williamson N, Mouradov D, Sieber O, Simpson R, Salim A, Bacic A. FunRich: a standalone tool for functional enrichment analysis. Proteomics. 2015;15(15):2597-601. DOI: 10.1002/pmic.201400515 PMID: 25921073

3. Snel B, Lehmann G, Bork P, Huynen M. STRING: a web-server to retrieve and display the repeatedly occurring neighbourhood of a gene. Nucleic Acids Research. 2000;28(18); 3442-3444. DOI: 10.1093/nar/28.18.3442 PMID: 10982861
